# Jak2 Tyrosine Kinase: A Potential Therapeutic Target for AT_1_ Receptor Mediated Cardiovascular Disease

**DOI:** 10.3390/ph3113478

**Published:** 2010-11-09

**Authors:** Annet Kirabo, Peter P. Sayeski

**Affiliations:** Department of Physiology and Functional Genomics, University of Florida College of Medicine, Gainesville, FL 32610, USA

**Keywords:** angiotensin II, Jak2, AT1-receptor, hypertension

## Abstract

Patients with hypertension often manifest a dysregulated renin-angiotensin-aldosterone system (RAAS). Most of the available treatment approaches for hypertension are targeted towards the RAAS including direct renin inhibition, ACE inhibition, angiotensin II type 1 receptor (AT_1_-R) blockade, and aldosterone receptor antagonism. The Jak2 signaling pathway is intricately coupled to the AT_1_-R signaling processes involved in hypertension. Here, we review the involvement of Jak2 in the pathogenesis of hypertension, and its potential as a therapeutic target for treatment of AT_1_-R mediated cardiovascular disease. Jak2 may provide a rational therapeutic approach for patients whose blood pressure is not controlled by standard therapies.

## 1. Introduction

Cardiovascular diseases are among the leading causes of death in the United States and other developed countries and hypertension is one of the major contributors to cardiovascular disease, end-organ damage, and death in the Western world [1]. The consequences of hypertension include myocardial ischemia, hypertensive heart disease, renal failure, peripheral atherosclerosis, and stoke. Central to these processes is the renin-angiotensin-aldosterone system (RAAS), which plays a major role in the pathophysiological processes leading to hypertension. 

Angiotensin II (Ang II) is the primary effecter hormone of the RAAS. There are two G protein-coupled receptor subtypes through which Ang II mediates its actions; the Ang II type 1 receptor (AT_1_-R) and Ang II type 2 receptor (AT_2_-R) [2,3]. Most of the physiological and pathophysiological cardiovascular actions of Ang II are mediated through the AT_1_-R [4,5]. The AT_2_-R is expressed at very high levels in the developing fetus, but its expression is very low in the cardiovascular system of adults [6]. Under normal physiological conditions, Ang II mediates responses that maintain electrolyte and blood pressure homeostasis. It affects glomerular blood flow via arteriolar vasoconstriction in the kidney and increases renal tubular sodium and water reabsorption by stimulating synthesis and secretion of aldosterone. In addition, Ang II stimulates release of vasopressin from the brain resulting in increased water retention. It also drives the thirst response. Finally, Ang II acts directly on vascular smooth muscle cells (VSMC) resulting in vasoconstriction and blood pressure regulation.

Perturbation of the RAAS is associated with the pathogenesis of a number of cardiovascular diseases. Ang II action via the AT_1_-R is particularly vital in the pathogenesis of cardiovascular disease resulting from hypertension. This is mainly due to its vasoconstrictive actions on VSMCs resulting in increased peripheral resistance and hypertension [6]. Ang II also acts on its receptors and mediates increased VSMC hyperplasia and hypertrophy, leading to increased peripheral vascular resistance. Most of the pathophysiologic effects result from chronic Ang II stimulation which elicits growth promoting effects leading to vascular disease [7]. Ang II infusion exacerbates neointima formation in animals with vascular balloon catheter injury [8], which is inhibited by RAAS blockers [9]. In addition, Ang II increases protein synthesis in VSMCs [10] and it stimulates growth in a number of cell types including VSMC, fibroblasts, adrenal cortical cells, cardiac myocytes, renal proximal tubular cells and tumor cells [11]. In cultured VSMCs, Ang II promotes hyperplasia, hypertrophy and migration [12,13,14,15]. It has also been implicated in inflammation, endothelial dysfunction, atherosclerosis, hypertension and renal fibrosis [16]. Chronic Ang II infusion in rodents induces VSMC proliferation in normal and injured vessels *in vivo* [8,17]. Interestingly, the growth factor-like Ang II-dependent responses are largely independent of its hemodynamic effects [18]. These studies suggest that Ang II acts as a growth factor under chronic exposure. However, the mechanisms that mediate the growth promoting effects of Ang II are still under scientific investigation. This review is aimed at analyzing the involvement of the tyrosine kinase, Jak2, in AT_1_-R mediated cardiovascular disease, and its potential as a treatment option for cardiovascular disease. 

## 2. The Janus Kinase Family of Proteins

There are four mammalian genes encoding the non-receptor *Janus* kinase (Jak) family of proteins; Jak1, Jak2, Jak3 and Tyk2 [19]. They contain seven regions with significant sequence homology and collectively, these regions are referred to as the Jak homology domains (JH1-JH7) [20]. The JH1 domain contains the tyrosine kinase domain, and is located within the carboxyl terminus of the protein. This domain binds ATP and harbors the phospho-transferase activity of the protein. The JH2 domain shows close homology to the JH1 domain, but lacks tyrosine kinase activity. It is therefore termed the pseudokinase domain. Acting *via* a cis mechanism, the JH2 domain negatively regulates the kinase activity of the JH1 domain [20,21]. The JH3 and half of the JH4 domain encode an SH2 like motif whose function is not well understood [22]. Finally, the remaining half of the JH4 domain, along with the entirety of the JH5, JH6, and JH7 domains, collectively encode the FERM domain. The FERM domain directly mediates the interaction of the Jak kinases with other cellular proteins such as cytokine receptors [23,24,25].

**Figure 1:**
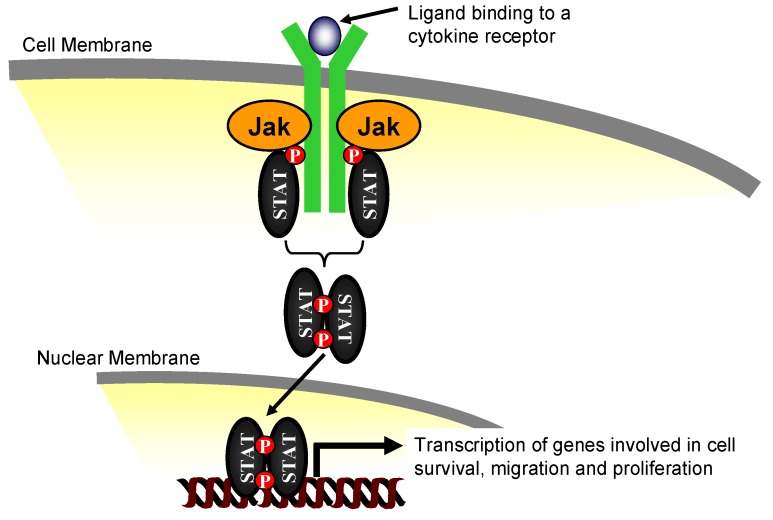
The Classical Jak/STAT Signaling Pathway. Ligand binding causes cytokine receptors to dimerize which results in Jak phosphorylation, recruitment of the Signal Transducer and Activator of Transcription (STAT) signaling proteins, which are then tyrosine phosphorylated by the Jaks. The phosphorylated STATs dimerize, and translocate into the nucleus where they bind to *cis-*inducible promoter elements to stimulate gene transcription.

The Jak kinases play a critical role in cytokine signaling. They transduce signals from the cell surface to the nucleus via the tyrosine phosphorylation of the Signal Transducers and Activators of Transcription (STAT) proteins. Phosphorylated STATs translocate into the nucleus where they bind to *cis-*inducible promoter elements and stimulate gene transcription (Figure 1). Insight into the *in vivo* function of each of the Jaks was gained via the generation of specific Jak kinase family knockout mice. Among the gene deletion models of the Jak family members, Jak2 deficient mice exhibited the most severe phenotype. Jak2 null mice die embryonically around day E12.5 of gestation due to impaired erythropoiesis and profound anemia [26,27]. These studies demonstrate that Jak2 is important in mouse development via erythropoietin receptor-dependent signaling. However, given the wide expression pattern of Jak2 in the body, there is still need to investigate its other biologically relevant functions as a mediator for cellular signaling in adult tissues. 

## 3. Jak2 in Angiotensin II-Induced Cardiovascular Disease

**Figure 2:**
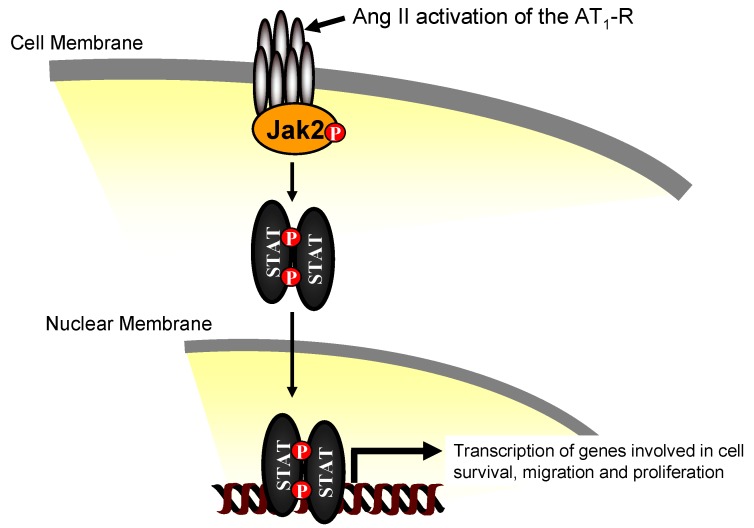
Activation of the Jak2 signaling cascade via the AT_1_-receptor results in mitogenic growth responses. Angiotensin II binding results in phosphorylation of Jak2. Active Jak2 recruits and phosphorylates STATs, which then dimerize, translocate into the nucleus, and mediate the transcription of genes involved in cell survival, migration, and proliferation.

Studies have shown that Ang II binding to the AT_1_-R triggers activation of Jak2, leading to intracellular signaling cascades in VSMCs and cardiac myocytes [28,29,30,31,32,33,34,35,36]. Ang II stimulates Jak2 co-association to the AT_1_-R in VSMCs leading to phosphorylation of Jak2 at Tyr 1007/Tyr 1008, phosphorylation of the STATs, and translocation of the STATs into the nucleus [29,37,38,39], resulting in cell growth/proliferative responses (Figure 2). Blockade of the RAAS by either angiotensin-converting enzyme (ACE) inhibitors or AT_1_-R specific antagonists prevents injury-induced neointima formation [9,40], and Ang II infusion exacerbates VSMC proliferation in arterial walls [8]. In addition, the genes of the RAAS are up regulated in neointima formation following vascular injury [41,42,43,44]. Interestingly, Jak2 has also been shown to play a role in other cardiovascular signaling processes [45]. For example, in VSMC, Jak2 plays a critical role in reactive oxygen species (ROS) dependent VSMC proliferation [46]. It is also involved in the pathogenesis of atherosclerosis via its interaction with cytokines such as interleukin 8 [47]. In addition, Jak2 activation has been linked to neointima formation and vascular occlusion in rat carotid arteries subjected to balloon injury, which is exacerbated by Ang II infusion [48].

Although it is well established that Jak2 interacts with the AT_1_-R resulting in cell growth and hypertrophy, there is no *in vitro* or *in vivo* evidence suggesting that the AT_1_-R mediated growth effects are exclusively through Jak2 activation. Further studies need to be done to establish the relative involvement of Jak2 activation in comparison to other pathways such as the mitogen-activated protein (MAP) kinase or pp60^c-src^ kinase in AT_1_-R mediated cardiovascular remodeling.

**Figure 3:**
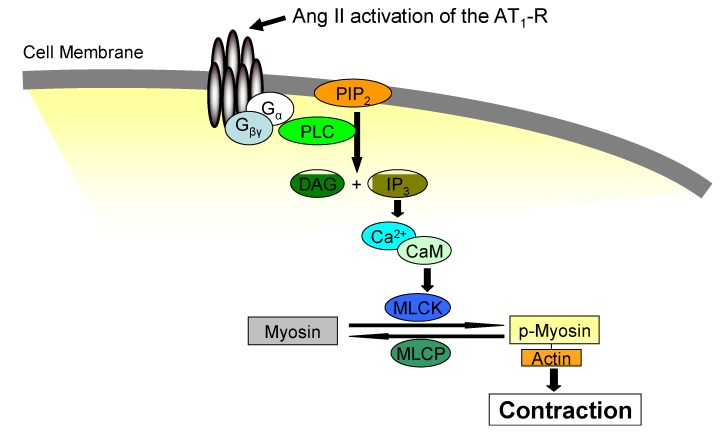
The mechanism by which Ang II mediates vasoconstriction. The binding of Ang II to the AT_1_-R activates the heterotrimeric G protein signaling pathway which leads to phospholipase C (PLC) activation. This releases inositol-1,4,5-triphosphate (IP_3_) and diacylglycerol (DAG) from phosphatidylinositol 4,5-bisphosphate (PIP_2_). IP_3_ binds to its receptor on the sarcoplasmic reticulum, allowing for Ca^2+^ efflux. Ang II also promotes an influx of external Ca^2+^ via calcium release activated calcium (CRAC) channels. Ca^2+^ binds to calmodulin and activates myosin light chain kinase (MLCK), which phosphorylates the myosin light chain and enhances the interaction between actin and myosin, resulting in enhanced vasoconstriction.

Jak2 not only mediates Ang II-dependent growth promoting effects, but is also involved in Ang II-induced contractile responses, increased vascular tone and hypertension. The established mechanism by which Ang II mediates vasoconstriction involves the heterotrimeric G protein-mediated pathway [49]. In VSMCs, the binding of Ang II to the AT_1_-R results in the activation of G_q_ [50] which leads to phospholipase C (PLC) activation. This releases inositol-1,4,5-triphosphate (IP_3_) and diacylglycerol (DAG) from plasma membrane derived phosphatidylinositol 4,5-bisphosphate [51]. Diacylglycerol stimulates protein kinase C (PKC) while IP_3_ binds to its receptor on the sarcoplasmic reticulum, allowing calcium efflux into the cytoplasm. Ang II also mediates an influx of external Ca^2+^ via calcium release activated calcium (CRAC) channels [52,53]. Ca^2+^ binds to calmodulin and activates myosin light chain kinase (MLCK), which phosphorylates the myosin light chain and enhances the interaction between actin and myosin, resulting in vasoconstriction [54]. The classical Ang II mediated signal transduction leading to vasoconstriction is summarized in Figure 3. 

Recently, Guilluy and colleagues demonstrated a role of Jak2 in the pathogenesis of hypertension [55]. The authors showed that Jak2 is involved in the Ang II-mediated activation of the Rho exchange factor, Arhgf1, resulting in enhanced vasoconstriction. It is not known whether the phosphorylation of Arhgef1 by Jak2 involves the Jak2 pool which is physically associated to AT_1_-R, or via an indirect mechanism.

ROS mediate signaling pathways involved in hypertension and vascular pathology [56,57] and Ang II is involved in mediating oxidative stress and oxidant signaling [54,58,59,60]. Many of the pathologic effects of Ang II in blood vessels are mediated by the generation of ROS via activation of NAD(P)H oxidases [56]. Ang II stimulates the activity of membrane-bound NAD(P)H oxidase in VSMCs and endothelial cells to produce ROS in the form of superoxide and hydrogen peroxide. Generation of such molecules causes vascular inflammation, fibrosis and endothelial dysfunction [16,56,61,62,63,64,65]. The Ang II-induced formation of ROS is not related to its hemodynamic effects as it does not occur in norepinephrine-induced hypertension [61,66]. Specifically, endothelial dysfunction was observed in rats made hypertensive by Ang II infusion, but not norepinephrine infusion. Furthermore, the endothelial dysfunction correlated positively with increased superoxide production in the arteries [61,66,67]. 

ROS have been shown to mediate RhoA/Rho kinase-induced Ca^2+^ sensitization in pulmonary vascular smooth muscle following chronic hypoxia [68]. Superoxide generated by Ang II inactivates nitric oxide (NO) in endothelial cells and VSMCs [69,70,71]. In addition, previous studies have shown that Rho kinase can be activated by increased ROS [68,72]. However, the mechanisms by which Ang II activates NAD(P)H oxidases to induce oxidative stress are still not well understood. A number of tyrosine kinases and phosphatases are known to be regulated by oxidative stress resulting in expression of inflammatory genes, endothelial dysfunction, VSMC growth, and extracellular matrix formation [56,58,61,62,73]. 

**Figure 4:**
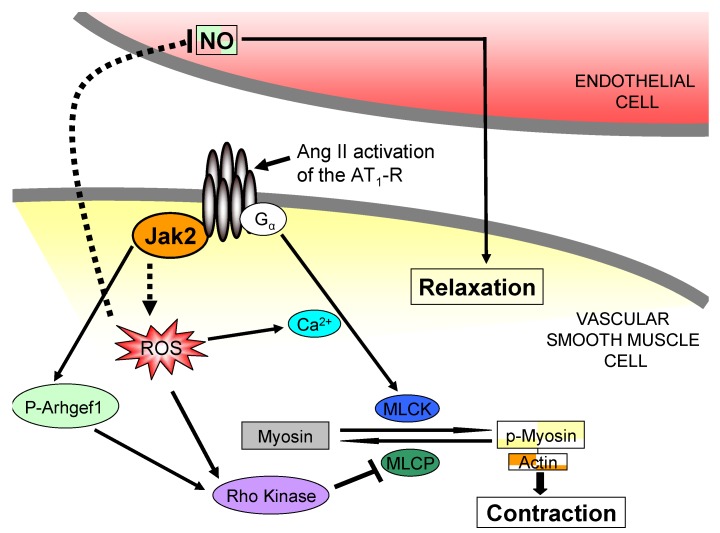
Proposed mechanisms through which Jak2 mediates Ang II-dependent vasoconstriction. Ang II binding to the AT_1_-R activates Jak2 (it is not known whether this involves the Jak2 pool physically associated with the AT_1_-R). Activated Jak2 phosphorylates Arhgf1 resulting in enhanced contraction via a Rho Kinase dependent mechanism. Jak2 is also believed to mediate intracellular increases in ROS. Higher levels of ROS increase intracellular Ca^2+^ sensitization, activate Rho Kinase, and scavenge endothelial nitric oxide all of which lead to enhanced VSMC contraction. The dotted lines indicate our current hypotheses on how Jak2 influences vascular tone.

There is evidence that Jak2 plays a critical role in mediating ROS dependent VSMC proliferation [46]. Activation of Jak2 results in higher levels of ROS and Jak2 inhibition leads to a dramatic reduction in oxidative stress [74]. Mutations which cause constitutive activation of Jak2, such as Jak2-V617F, increase the levels of ROS within cells, and inhibition of Jak2 leads to reduction of ROS in these same cells [74,75]. Hence, production of ROS by the AT_1_-R, and Jak2 activation have been experimentally demonstrated. However, it is still not known whether Jak2 mediates Ang II induced production of ROS via the AT_1_-R. There is still a need to elucidate the specific mechanisms by which Jak2 contributes to production of ROS, and whether it plays a role in regulating NO availability by contributing to superoxide production. Furthermore, it is not known whether Jak2 can activate Rho kinase via ROS-dependent mechanisms. The proposed mechanisms through which Jak2 mediates vasoconstriction are represented in Figure 4.

## 4. Pharmacological Jak2 Inhibition: An Emerging Therapeutic Strategy in Jak2-Mediated Diseases

Jak2 kinase function is critical for normal hematopoietic growth factor signaling [76]. On the other hand, hyper-kinetic Jak2 tyrosine kinase signaling causes several hematologic diseases including some forms of leukemia, lymphoma, and myeloma. Gain-of-function somatic mutations in the Jak2 allele are also known to be a causative agent in the pathogenesis of the myeloproliferative neoplasms (MPN) [77]. MPNs are clonal disorders of multipotent hematopoietic progenitors characterized by increased hematopoiesis. They include polycythemia vera (PV), essential thrombocythaemia (ET) and primary myelofibrosis (PMF). MPNs have a relatively high prevalence with the number of cases ranging from about 130,000 to 150,000 in the United States alone [78]. 

The clinical symptoms of MPNs include bleeding, thrombosis, splenomegaly, progressive bone marrow failure, and a propensity for malignant transformation in the form of acute myeloid leukemia. One Jak2 mutation which causes MPNs is a valine to phenylalanine substitution at residue 617 (Jak2-V617F) within the pseudokinase domain. This mutation relieves the inhibitory potential that the JH2 domain normally exerts on the JH1 kinase domain and the consequence of this lost inhibitory potential is constitutive activation of the Jak2 signaling pathway [79,80,81,82,83]. The Jak2-V617F mutation has also been implicated in other Jak2 mediated human diseases such as chronic myelomonocytic leukemia, myelodysplastic syndrome, systemic mastocytosis, chronic neutrophilic leukemia, and acute myeloid leukemia [84,85,86]. 

Based on the identification of activating Jak2 mutations in MPNs, great effort has been aimed at developing inhibitors that target Jak2 [87]. Accordingly, a number of small molecule Jak2 inhibitors, which have potential therapeutic efficacy against Jak2-mediated disorders, have been developed [88,89,90,91,92,93,94,95,96]. These compounds inhibit the pathologic cell growth and signaling in cell lines transformed by Jak2 mutations *in vitro*, in murine models *in vivo*, and in bone marrow samples obtained from MPN patients and cultured *ex vivo* [88,96,97,98]. While some of these compounds are in pre-clinical stages of development [99], others are currently in clinical trials for the treatment of MPNs [88,100,101]. Early reports from these studies indicate that direct inhibition of Jak2 with small molecule inhibitor therapy improved some clinical measures such as spleen size and certain blood counts [100]. Side effects associated with Jak2 inhibitor therapy included fatigue, neurotoxicity, and gastrointestinal disturbances [100]. However, given the existing correlation between Jak2 kinase activity and cardiovascular disease, perhaps changes in blood pressure or other cardiovascular readouts should be followed in these patients. 

## 5. Jak2 Inhibitors and Their Potential for Cardiovascular Disease Therapy

The pathogenesis of hypertension often involves a complex combination of causes including genetic and environmental factors [102]. The current pharmacological treatments for hypertension are mainly targeted towards inhibition or prevention of action of vasoconstrictor hormones including Ang II. Treatment of resistant hypertension currently entails choosing medications with complementary mechanisms of action such as optimizing diuretic use, and/or mineralocorticoid antagonism [103]. However, due to the multifactorial nature of the disease pathogenesis, there are still subsets of patients in whom available treatments are increasingly becoming ineffective. Treatment resistant hypertension presents an increasing dilemma in the clinical setting, and patients with resistant hypertension have increased cardiovascular risk [103]. Therefore, there is still need to identify other genetic targets, to provide more individualized treatments for such patients. In addition, a number of patients are non-responsive to mono-antihypertensive therapy and there is often need to use combination therapy [104].

Since Jak2 has been shown to regulate Ang II-mediated signaling downstream of the AT_1_-R, it may represent a valuable new target for anti-hypertensive therapeutic strategies. AG490, a tyrphostin well known for inhibiting Jak2 [105] has been shown to prevent hypertension [55], and neointima formation [48] in animal models. As such, these studies implicate Jak2 as an important modulator of blood pressure and cardiovascular disease. Therefore, therapeutic approaches using inhibition of Jak2 to regulate Ang II-mediated AT_1_-R stimulation is an intriguing novel target for the treatment of hypertension. This implies a potential new therapeutic target for multidrug resistant hypertension. The realization of therapeutic benefit from Jak2 inhibition in cardiovascular diseases may entail identifying optimized inhibitors with unique profiles to maximize therapeutic potential. In addition, given the potential side effects that may arise from Jak2 inhibition, there is need for a risk/benefit assessment of using Jak2 as a target in treatment of cardiovascular disease. Being a downstream signaling molecule of the AT1-R, Jak2 is well positioned, and may offer a new, more specific target in the treatment of Ang II-mediated cardiovascular diseases. Ang II acts on the AT_1_-R resulting in the stimulation of multiple downstream signaling cascades, leading to various effects. Since Jak2 is a downstream signaling molecule of the AT_1_-R, it may present a more specific target for treatment of cardiovascular disease while avoiding side effects arising from inhibition of non diseased signaling pathways. 

## 6. Conclusions

Hypertension is a complex and multi-factorial disease and is unlikely to be successfully treated with a single approach. Current standard therapies include inhibition of the RAAS as well as enhancement of nitric oxide-mediated relaxation of VSMC. Jak2 is centrally located as a downstream signaling molecule of the AT_1_-R and it is involved in many of the signaling cascades regulated by Ang II including oxidative stress. It appears that in a multi-factorial disease such as hypertension, Jak2 may provide a more specific target as a therapeutic approach. Jak2 inhibition offers a potential base for further study and development of therapeutic options to overcome cardiovascular disease and ultimately, may be considered as an adjunct or alternative to current therapeutic agents. 
